# Tpeak-Tend/QT interval predicts ST-segment resolution and major adverse cardiac events in acute ST-segment elevation myocardial infarction patients undergoing percutaneous coronary intervention

**DOI:** 10.1097/MD.0000000000012943

**Published:** 2018-10-26

**Authors:** Xianpei Wang, Lu Zhang, Chuanyu Gao, Jialu Zhu, Xiaohang Yang

**Affiliations:** Department of Cardiology, People's Hospital of Zhengzhou University (Henan Provincial People's Hospital), Fuwai Central China Cardiovascular Hospital, Zhengzhou, Henan Province, China.

**Keywords:** major adverse cardiac events, myocardial infarction, ST-segment resolution, Tp-e interval, Tp-e/QT ratio

## Abstract

Elevated ST-segment and increased Tpeak-Tend interval (Tp-e) were prognostic predictors in major adverse cardiac events (MACEs) in ST-segment elevation myocardial infarction (STEMI). The electrophysiologic relationship between them during percutaneous coronary intervention (PCI) needs to elucidate.

Patients with STEMI admitted to hospital were prospectively evaluated. ST-segment resolution (STR) (defined as ≥50% reduction as the complete-STR [CSTR] group, <50% as incomplete-STR [ISTR] group), Tp-e interval, and ratio of Tp-e to QT interval (Tp-e/QT) were measured, calculated and analyzed with MACEs.

Tp-ec interval (corrected Tp-e interval, *P* < .001) and Tp-e/QT ratio (*P* < .001) were significantly increased by myocardial infarction and partly recovered post-PCI. Patients with ISTR showed more increased Tp-ec interval (*P* < .001) and Tp-e/QT ratio (*P* < .001) than those in CSTR groups post-PCI. In multivariate analysis and receiver operating characteristic curves analysis, Tp-e/QT was an independent and strongest predictor for STR. STR and electrocardiogram parameters with a cutoff value for predicting STR showed prognostic value for MACE in STEMI in Kaplan–Meier survival analysis.

Both STR and change of Tp-e parameters were not only predictors of arrhythmia, but also prognostic factors of MACE in patients with STEMI after PCI.

## 1 Introduction

Elevation of the ST segment in the electrocardiogram (ECG) is a classical hallmark of acute transmural myocardial infarction, which is also the major clinical criterion for deciding which patients with chest pain required emergent coronary revascularization. Numerous studies have reported that the degree of resolution of ST-segment resolution (STR) following reperfusion therapy in patients with ST-segment elevation myocardial infarction (STEMI) represents a predictor of the preserved left ventricular function, malignant ventricular arrhythmia or sudden cardiac death, and long-term survival.^[1]^ Reperfusion therapy was thought to improve prognosis, mainly through recanalization of the epicardial vessels; however, in recent years, studies have shown that even in cases in which adequate epicardial vessel recanalization was achieved, myocardial tissue perfusion might still be insufficient in the presence of myocardial microcirculation disturbance, and STR was incomplete in ECG leads.^[1–3]^ In ischemic cellular models, the extent of elevated ST-segment represented phase 2 reentry, which is the primary mechanism of arrhythmogenesis in patients with Brugada syndrome (Brs) and STEMI, who were characterized by increased Tpeak-Tend (Tp-e) interval (defined as the interval from the peak to the end of the T-wave) in ECG leads.^[4–6]^

Some reports have established that Tp-e interval was prolonged in patients with STEMI and that increased interval predicted the all-cause mortality, but the parameters were obtained in noninfarct-related leads.^[6,7]^ The mechanism for the genesis and utility of the Tp-e interval remains controversial.^[8]^ The data derived from isolated wedge preparation suggest that Tp-e interval was an index of transmural dispersion of repolarization (TDR). While the alternative view speculated that the Tp-e interval reflected the global dispersion of repolarization.^[8,9]^ However, increased Tp-e interval, accompanied with elevated ST-segment in precordial lead ECG, was considered a risk factor for malignant ventricular arrhythmia in patients with Brs.^[4]^ Therefore, we hypothesized that elevated ST segment and prolonged Tp-e interval were mechanism-related in ECG, as those in Brs, and had similar prognostic value in patients with STEMI undergoing reperfusion of percutaneous coronary intervention (PCI). Further, elucidating the relationship of STR and Tp-e interval in patients with STEMI might increase understanding of the origin and function of the Tp-e interval and provide coordinated prognostic value for major adverse cardiac events (MACEs). So this study was to elucidate electrophysiologic relationship of elevated ST-segment and Tp-e interval and their prognostic values on MACE in STEMI during PCI.

## 2 Methods

### 2.1 Study subjects

Patients were recruited between January 2012 and June 2015 from People's Hospital of Zhengzhou University, Zhengzhou, China. The 374 consecutive patients with the first attack of STEMI seen <12 hours after the onset of symptoms were included if they were having typical chest pain lasting >30 minutes, with ≥0.1 mV ST-segment elevation in ≥2 limb leads or ≥0.2 mV ST-segment elevation in 2 contiguous precordial leads, and elevation of the serum troponin I levels greater than the upper limit of normal were enrolled. All patients underwent angiography and showed confirmatory angiographic evidence of total occlusion, that is, TIMI (thrombolysis in myocardial infarction) grade 0 or 1 flow, of the infarct-related coronary artery. All subjects then underwent PCI and only patients in whom TIMI grade 3 flow was restored were selected for the final study. The exclusion criteria were as follows: left bundle branch block, right bundle branch block, and ventricular pacing; cardiac pump failure at the time of cardiac echo study; and significant restenosis of infarct-related arteries as confirmed on follow-up coronary angiography. After pPCI, patients received usual standard of care according to clinical guidelines, including treatment with clopidogrel for 12 months.

A total of 58 patients were excluded from this study for the following reasons: persistent TIMI grade 2 flow after PCI (7 patients), uninterpretable ECGs due to the development of left bundle branch block (5 patients), and unmeasurable Tp-e interval because of monophasic curve of elevated ST-segment (46 patients). The study was conducted with the approval of the ethics committee of People's Hospital of Zhengzhou University, and written informed consent was obtained from all the patients.

### 2.2 Angiography and PCI

All patients received heparin (5000 U) before angiography. Coronary angiography was performed using the standard Judkins technique. Before PCI, an additional dose of heparin (120 U/kg) was administered intravenously. Coronary balloon angioplasty or coronary stent implantation was performed in all patients. Angioplasty was considered successful when the residual stenosis was reduced to <25%, with TIMI grade 3 flow. Patients who underwent coronary stent implantation were initiated on antiplatelet drug therapy with clopidogrel (75 mg once daily) plus aspirin (100 mg once daily).

### 2.3 Electrocardiographic analysis

The ECG was recorded with a standard digital recorder as 12 simultaneous leads at a paper speed of 25 mm/s. It was performed at baseline (first ECG) and at 100 minutes after PCI (second ECG), as described previously.^[10]^ The single ECG lead with maximum ST-segment elevation at that time point was considered. The degree of STR was assessed by the reduction in the ST-segment elevation of the same lead between the first and second ECG and expressed as a percentage. Patients with ≥50% reduction of the ST-segment elevation on the second ECG were classified into the complete-STR (CSTR) group, while the remaining patients were classified into the incomplete-STR (ISTR) group.^[1,10]^ Although all patients showed restoration of TIMI 3 grade flow, 222 of the 316 patients (70.3%) were classified into the CSTR group, while the remaining patients (94 of 316, 29.7%) were classified into the ISTR group. Both groups had normal results on physical examination and no history of cardiac disease, chronic obstructive lung disease, renal disease, or endocrine disorders. No patient was receiving antiarrhythmic agents or other types of therapy that could affect the ECG parameters.

The QT and Tp-e were manually measured according to the procedure in our paper.^[11]^ The corrected QT (QTc) and corrected Tp-e (Tp-ec) were obtained using Bazett formula. QT interval was measured from the onset of the QRS to the end of the T-wave, defined as a return to the T-P baseline. Leads with T-wave amplitude <1.5 mm were excluded from the analysis. The maximum of QTc in all leads was applied to the analysis. The QT dispersion was defined as the difference between the maximum and minimum QT interval of the 12 leads. Because of the dispersion of Tp-e interval between infarction-related and noninfarction-related leads, the maximum of Tp-e in all measured leads was in infarction-related lead and entered into the analysis. The Tp-e interval was obtained from the peak of the T-wave to the end of T-wave in ST-segment elevated leads. In the case of negative or biphasic T-waves, QTpeak was measured to the nadir of the T-wave. If elevated ST-segment and T-waves of ECG were fused into monophasic curve, the cases (46 patients) were excluded from the study. The measurement of each parameter was obtained by averaging 3 consecutive beats. Two independent experts obtained the measurements and in case of a difference of 20 milliseconds in each measurement, the 3rd expert was recruited. The interobserver Pearson correlation coefficients of QTc dispersion, Tp-ec, and Tp-e/QT were 0.959, 0.977, and 0.982, respectively.

### 2.4 MACE evaluation and follow-up

The primary endpoint was the prevalence of MACEs, defined as a composite of cardiac death, malignant arrhythmia event (defined as ventricular tachycardia/ventricular fibrillation, syncope, aborted sudden death), at 1-year follow-up. If a patient experienced more than one event, the first event was chosen for the combined clinical endpoint. Patients were considered at risk from the time of admission for the treatment of STEMI.

### 2.5 Statistical analysis

Continuous variables are expressed as mean ± standard deviation and, if appropriate, were compared using the dependent-samples Student *t* test. Categorical variables are expressed as numbers and percentages and, if appropriate, were compared with the Chi-squared test. Binary logistic regression was performed to assess the association of each predictor with outcome and further multivariable logistic regression analysis was conducted to estimate the association of Tp-e parameters with MACE after controlling for the other factors. Analyses of receiver operating characteristic (ROC) curves were made to examine prognostic value of ECG parameters and determine cutoff values. The optimal cutoff value was defined as the value yielding the maximal Youden index (Youden index = Max ([sensitivity] + [specificity] − 1)), or the best combined sensitivity and specificity.^[12,13]^ Kaplan–Meier plots with log-rank test were performed to analyze correlation of STR and ECG parameters with combined MACE. Statistical analysis was performed using SPSS 20.0.0 (IBM Inc, Armonk, NY). Area under ROC curve (AUC) comparison was performed by MedCalc15.2.2 (MedCalc Software, Ostend, Belgium) with *Z* test. A *P*-value <.05 was deemed statistically significant.

## 3 Results

### 3.1 Clinical characteristics

Demographic, clinical, and angiographic data of CSTR (n = 222) and ISTR (n = 94) groups are summarized in Table 1. No patients were lost to follow-up. Stent implantation was performed in 294 of the 316 patients (93%). Myocardial blood supply was restored to TIMI 3 at an average of 652 ± 321 minutes after onset. The second ECG was obtained at an average of 108 ± 32 minutes after reperfusion. Between the 2 groups, there were no significant differences with respect to age, sex, and cardiovascular disease risk factors, such as arterial hypertension, diabetes mellitus, dyslipidemia and tobacco smoking, and medication use. There were also no significant differences between the 2 groups regarding culprit lesion and revascularization time from symptom onset (all *P* > .05). The patients were given standard secondary prevention treatment according to guideline and there were no significant differences in treatment options between CSTR and ISTR groups (*P* > .05).

**Table 1:**
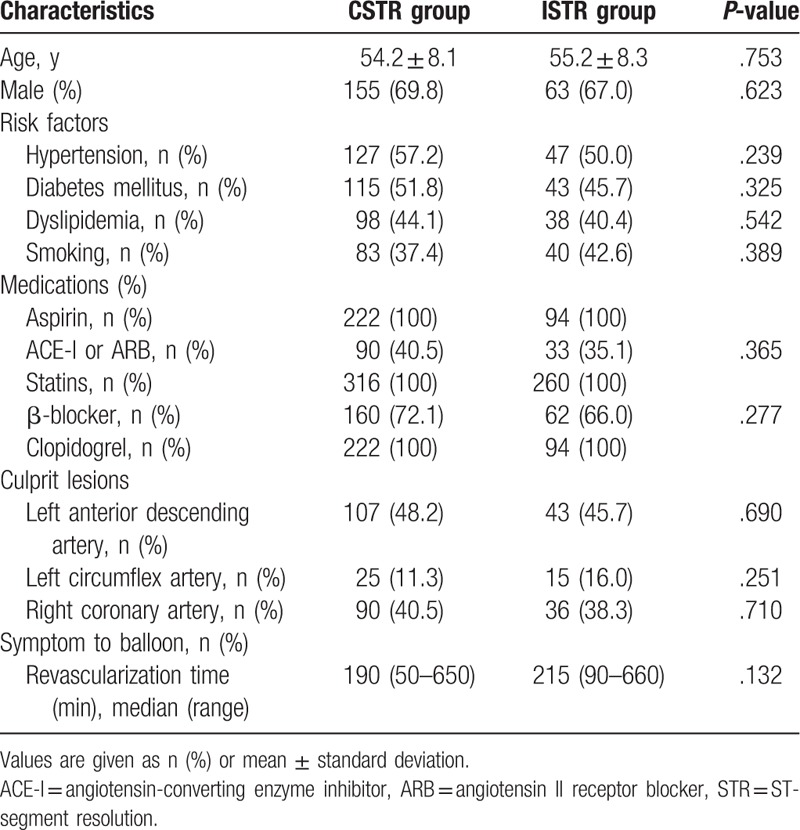
Baseline characteristics of the patients (n = 222 in complete-STR and n = 94 in Incomplete-STR).

### 3.2 Comparison of ECG parameters in STEMI patients pre- and post-PCI

The cases with STEMI were administrated ECG recorded in 12 hours after myocardial infarction and in 100 minutes after PCI. QTc, QTc dispersion, Tp-ec, and Tp-e/QT were measured and calculated in ECG leads. QTc interval (436 ± 32.9 pre-PCI vs 402 ± 30.1 post-PCI, *P* = .042), QTc dispersion (92 ± 23.5 pre-PCI vs 68 ± 18.4 post-PCI, *P* < .001), and Tp-ec interval (159 ± 26.9 pre-PCI vs 126 ± 22.5 post-PCI, *P* < .001) were prolonged by myocardial infarction and significantly partly recovered after PCI. Revascularization therapy of PCI partly recovered significantly increased Tp-e/QT ratio (0.366 ± 0.09 pre-PCI vs 0.292 ± 0.08 post-PCI, *P* < .001) (Table 2).

**Table 2:**
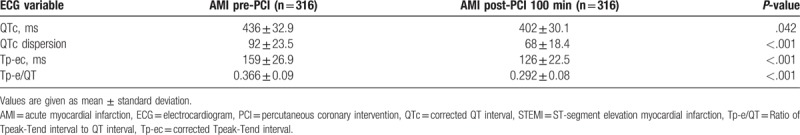
QTc, Tp-ec, Tp-e/QT in infarction-related lead pre- and post-PCI in patients with STEMI.

### 3.3 Comparison of changes of ECG parameters between CSTR and ISTR groups in STEMI patients

The correlation coefficient of maximum of ST-segment elevation with corresponding Tp-ec and Tp-e/QT was 0.798 (*P* < .01) and 0.823 (*P* < .01), respectively, in STEMI patients pre-PCI. Patients with ISTR showed more prolonged QTc dispersion (78 ± 19.6 vs 58 ± 16.7; *P* = .038), Tp-ec interval (157 ± 29.9 ± 29.9 vs 128 ± 24.2; *P* < .001), and greater Tp-e/QT ratio (0.337 ± 0.07 vs 0.275 ± 0.06; *P* < .001) than those in the CSTR groups after PCI (Table 3).

**Table 3:**
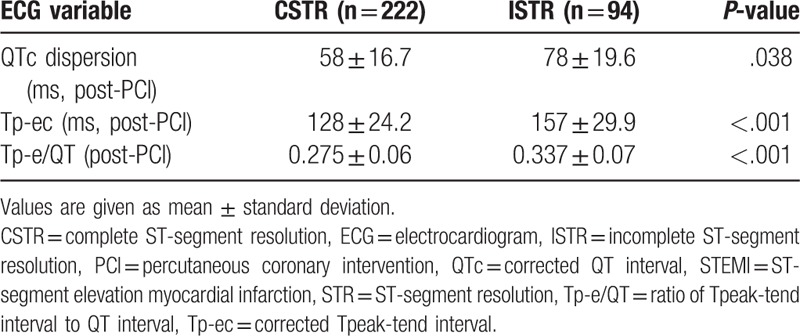
Comparison of change of ECG parameters between complete STR and incomplete STR groups in STEMI patients (mean ± standard deviation).

### 3.4 Univariate and multivariate regression model for predicting STR

A multiple collinearity diagnosis was implemented among QT dispersion, Tp-ec interval, and Tp-e/QT ratio of post-PCI. The maximum value of variance inflation factor was 7.6, and the maximum value of condition index was 25.9, which meant the existence of certain collinearity among these ECG parameters. Then the ECG parameters were tested on the univariate binary logistic regression analysis, as shown in Table 4. ISTR was defined as malignant events of dependent variables. QTc dispersion (odds ratio [OR] = 0.804; 95% confidence intervals [CI] 0.326–1.030; *P* = .114) were not significantly associated with STR. Increased Tp-ec (OR = 0.645; 95% CI 0.405–0.890; *P* = .043) and Tp-e/QT (OR = 0.538; 95% CI 0.326–769; *P* = .039) post-PCI inclined to predict ISTR, or both of them were negatively correlated with STR. In the following, stepwise multivariate analysis was performed. Only Tp-e/QT ratio entered into the model. Both OR and CI were constant in the model (Table 4). Therefore, Tp-e/QT ratio of post-PCI remained the strongest predictor of STR.

**Table 4:**
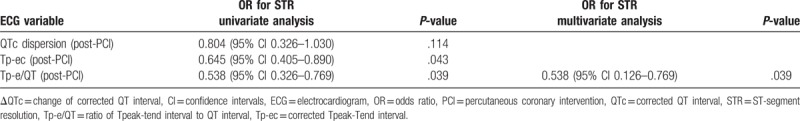
Univariate and multivariate regression model for predicting STR.

### 3.5 ROC curve and cutoff value of ECG parameters for predicting STR

The ROC curve analysis was performed to assess the sensitivity, specificity, and best cutoff values of different ECG parameters for the prediction of STR. The AUC of ROC was 0.692 (95% CI 0.552–0.838) for Tp-ec, and 0.883 (95% CI 0.626–0.957) for Tp-e/QT ratio. Comparisons of AUC with *Z* test using MedCalc software showed that AUC of Tp-e/QT was significantly different with that in Tp-ec interval (*P* < .05). The best cutoff value, or best combination of sensitivity and specificity, was found for Tp-ec ≥139 milliseconds (90% and 65%), and Tp-e/QT ≥0.31 (83.6% and 95%), respectively. The results of comparison between AUC of ROC of ECG parameters indicated that Tp-e/QT was independent predictors for STR in patients undergoing PCI for STEMI. Combined with the results of binary logistic regression analysis, Tp-e/QT ratio of post-PCI was the best discriminator to predict STR.

### 3.6 STR and ECG parameters with cutoff value for predicting STR predicted MACE during one-year follow-up

**Figure 1:**
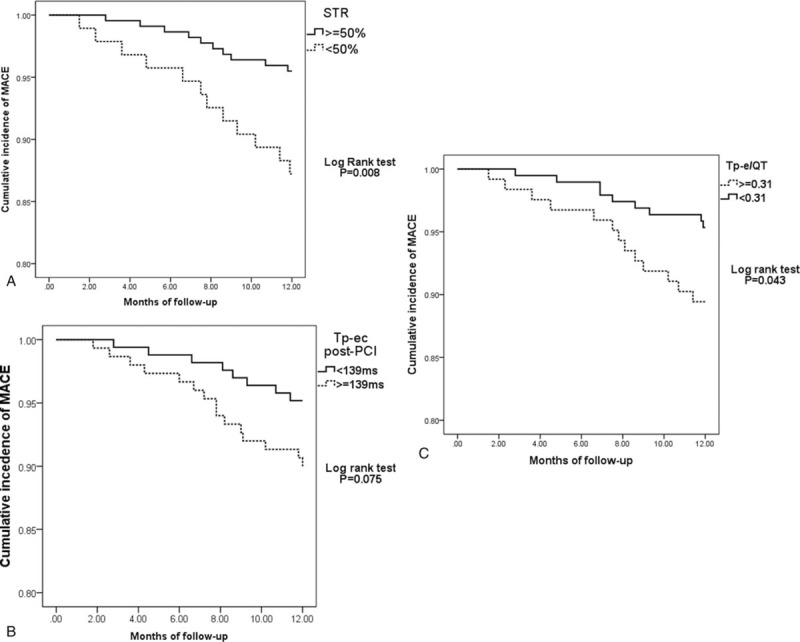
Kaplan–Meier curve of the cumulative incidence of major adverse cardiac event (MACE) during 1-year follow-up for complete ST-segment resolution (STR) and incomplete STR (A), Tp-ec post-PCI ≥139 milliseconds group and that <139 milliseconds (B), Tp-e/QT ≥0.31 group and that <0.31 (C). A log-rank test was used to calculate the *P-*value.

It was analyzed by Kaplan–Meier curves for correlation of STR and ECG parameters with cutoff value for predicting STR, with MACE during 1-year follow-up to elucidate whether cutoff value of ECG parameters for predicting STR could also possess prognostic value on MACE. The cumulative incidence of the MACE was plotted with STR at a cutoff value of 50%, Tp-ec post-PCI at 139 milliseconds, Tp-e/QT post-PCI at 0.31, respectively (Fig. 1). By 1-year, 10 of 222 (4.5%) subjects in the CSTR group suffered from MACE, which had significantly lower event rates than with those in the ISTR group (12 of 94 patients [12.8%] (*P* = .008, Fig. 1A). The cumulative incidence of MACE was 6.5% (10 of 155) in Tp-ec <139 milliseconds group post-PCI and 10.8% (15 of 139) in Tp-ec ≥139 milliseconds group. There was no significant difference between these 2 groups (*P* = .075, Fig. 1B). In the Tp-e/QT ≥0.31 group, the cumulative incidence of MACE was 10.6% (13 of 123 patients), which was significantly higher than that in Tp-e/QT <0.31 group (4.7%, 9 of 184) (*P* = .043, Fig. 1C). STR and Tp-e/QT with cutoff value for predicting STR, had the prognostic value on MACE in patients undergoing PCI for acute STEMI.

## 4 Discussion

This study investigated the relationship between STR and Tp-e-related ECG parameters in infarction-related leads in patients with STEMI undergoing PCI and had the following main findings: QTc, Tp-ec interval, QTc dispersion, and Tp-e/QT ratio were significantly increased by coronary artery occlusion, while reperfusion therapy could partly recover these values; there were greater QTc dispersion, Tp-ec interval, and Tp-e/QT ratio post-PCI in the ISTR group than those in the CSTR group; increased Tp-ec interval and Tp-e/QT ratio post-PCI predicted less STR. Tp-e/QT ratio was the strongest predictor for STR; STR and Tp-e/QT ratio with cutoff value for predicting STR had the prognostic value on MACE in patients with acute STEMI undergoing PCI. Therefore, elevated ST-segment was mechanism-related with Tp-e interval in STEMI.

In this study, QT and Tp-e interval were measured in infarction-related and noninfarction-related ECG leads. QT interval has been shown to represent an approximate measure of ventricular repolarization. Spatial variability in the QT intervals of ECGs (QT dispersion) has been shown to reflect variability of ventricular repolarization dispersion and was a marker of arrhythmogenic potential in variant angina and STEMI.^[14,15]^ In this investigation, QT dispersion was increased by myocardial infarction, and there was greater QT dispersion in the ISTR group than in the CSTR group. The existence of QT dispersion reflected inhomogeneity in ventricular repolarization in infarcted region and noninfarcted region. However, QT dispersion could not predict STR in univariate analysis. Tp-e interval was the main component reflecting repolarization discrepancy of QT interval. So Tp-e interval was more valuable than QTc and QT dispersion as a prognostic factor of ventricular repolarization dispersion and MACE in patients with acquired long QT syndromes (LQTS),^[9]^ Brs,^[4]^ and STEMI,^[6,14]^ just as that in our study. However, Tp-e interval was investigated in noninfarction-related leads in patients with STEMI because of the difficulty in assessing T-wave markers.^[6,14]^ Furthermore, Tp-e dispersion was significantly correlated with occurrence of life-threatening arrhythmic events in Brs and vasospastic angina (VA),^[4,11]^ which established that there was Tp-e dispersion between elevated ST-segment and nonelevated ST-segment leads in 12-lead ECG in patients with Brs and VA. There was also Tp-e dispersion in different leads of patients with STEMI in this study, so ECG parameters of infarction-related leads, not noninfarction-related leads, more truly reflected the change caused by a pathophysiologic condition, such as artery occlusion. Therefore, the relationship of elevated ST-segment and Tp-e parameters was investigated in infarction-related leads, and the maximum of ECG parameters in all leads was applied to the analysis in this study.

Malignant ventricular arrhythmia was a reason for poor prognosis for acute STEMI. Tp-ec or Tp-e/QT ratio served as a sensitive index of arrhythmogenesis because it provided an estimate of dispersion of repolarization and predicted occurrence of arrhythmic events in LQTS, Brs, and short QT syndrome and in patients with organic heart disease such as VA and acute myocardial infarction.^[6,11,14,16–18]^ Our study showed Tp-ec interval and Tp-e/QT ratio in the ST-elevation leads were significantly increased. In our other study, we had discussed that TDR played a significant role on the constituent of Tp-e interval in pathophysiologic conditions, such as VA, regardless of which theory was employed for Tp-e interval in normal condition.^[11]^ Therefore, increased Tp-e interval caused by coronary artery occlusion provided a substrate for the prolongation of TDR and was correlated with STR and MACE of STEMI patients in our study.

**Figure 2:**
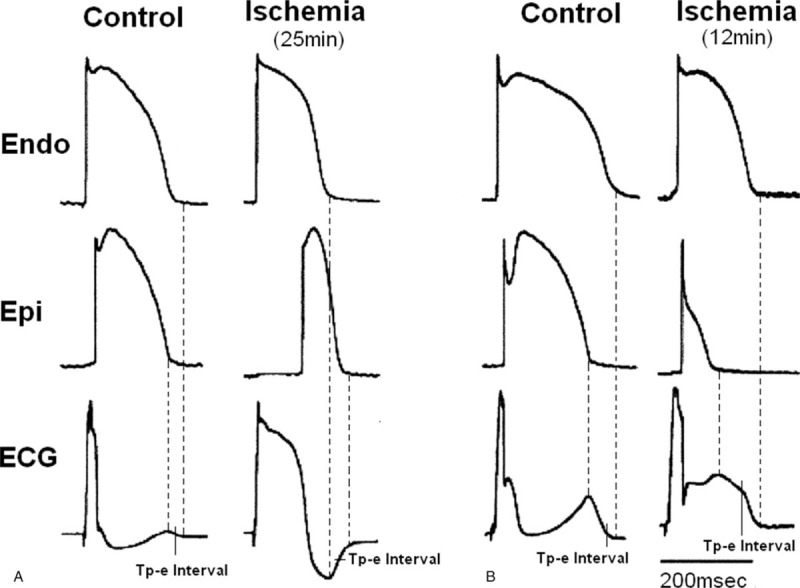
Electrophysiologic effect of ischemia in the ventricular wedge model. Results were from 2 different preparations. (A) Recordings obtained under control conditions and after 25 minutes of ischemia. Tp-e was increased significantly in ischemia condition compared with control. (B) Recordings obtained under control conditions and after 12 minutes of ischemia. Tp-e was also increased significantly in ischemia condition compared with control. BCL = 800 milliseconds (modified from Ref. 19, with permission).

In Brugada syndrome, the resultant transmural voltage gradients induced by depression or loss of the AP dome occurring in ventricular epicardium caused an ST-segment elevation, which predisposed the ventricle to the development of phase 2 reentrant extrasystoles, then precipitated VT/VF. Meanwhile, the Tp-e interval increased significantly leading to amplification of Tp-e/QT ratio in right precordial leads with coved configuration of ST elevation and was considered as risk factors for arrhythmic events in Brugada syndrome patients. These results indicated that dispersion of repolarization including epicardial and transmural dispersion underlay the phase 2 reentry, and therefore induced VT/VF. Tp-e interval then became an index of TDR, reflecting partly potential gradient of phase 2 reentry in Brugada syndrome patients.^[4]^ In this study, the amplitude of ST-segment elevation was positively correlated with corresponding Tp-ec and Tp-e/QT pre-PCI. Increased Tp-ec interval and Tp-e/QT ratio post-PCI were negatively correlated with STR in STEMI. If Tp-e interval was considered a substrate of TDR in STEMI, we could describe this relationship with the following scheme (Fig. 2, modified from Ref. 19, with permission of Copyright © 2014 Elsevier Inc).^[19]^ Transmembrane action potentials (APs) from Endo and Epi and the ECG were simultaneously recorded from top to bottom. Figure 2A illustrates that an apparent ST-segment elevation (the difference of Endo AP plateau [or R-wave in this paper] and delayed Epi AP plateau) and inverted T-wave was induced by ischemia-induced transmural conduction slowing (slowing of Epi conduction). Simultaneous ECG recordings showed Tp-e interval (the difference of end of Endo AP to the end of delayed Epi AP) was increased significantly in ischemia condition compared with that in control after 25 minutes of ischemia in ventricular wedges. If elevated ST-segment was considered a marked prolongation of the R-wave of ECG because of delayed Epi transmural conduction, the extent of ST-segment elevation was determined by the decreased extent and amplitude of delayed conduction, which also decided the longitude of Tp-e interval. Figure 2B shows that an all-or-none repolarization at the end of phase 1 of AP was induced by the ischemia-induced loss of the epicardial AP dome after 12 minutes of ischemia, which produced the voltage gradients at the AP plateau level between the endocardial and epicardial regions and then induced nonisoelectric ST-segment elevation, while the potential difference between Epi and Endo determined the TDR, or the duration of Tp-e interval. Therefore, ischemia induced the potential gradients of transmural phase 2 plateau of AP, which induced the elevation of ECG ST-segment and the difference augmentation of APD; the latter induced the increased Tp-e interval. If epicardial AP disappeared, ECG showed as monophasic curve, just like the shape of endocardial APD. Reperfusion could partly recover these gradients and resulted in the STR and decreased Tp-e interval in acute ischemia condition. These mechanisms agreed with the results on the relationship between STR and investigated ECG parameters in this study.

In this study, Tp-e/QT ratio was a relatively sensitive factor to predict STR compared with Tp-ec. Tp-e/QT seemed to represent a more sensitive predictor of arrhythmogenesis than Tp-e, as Tp-e/QT ratio provided an estimate of the repolarization dispersion relative to the total duration of repolarization, avoiding possible interference effects of heart rate variability and inter-individual variation of QT interval.^[18]^ Some investigations have reported that STR and Tp-e/QT predicted MACE in STEMI, respectively.^[1,6]^ While STR and Tp-e parameters with cutoff value for predicting STR had the prognostic value for MACE in patients undergoing PCI for acute STEMI in this study, meant STR and Tp-e parameters were mechanism-related such as in the above scheme model. The extent of ST elevation could represent the transmural potential gradients of phase 2 reentry.^[5,20]^ Increased Tp-e interval could facilitate the after-depolarization (early after-depolarization and delayed after-depolarization)-induced triggered activity and following arrhythmia, similar to the mechanisms of arrhythmogenesis in LQTS conditions.^[21]^ Therefore, increased Tp-e interval and Tp-e/QT ratio, indicated the augmentation of TDR and trigger activity, could be one of the mechanisms of the onset of arrhythmia in STEMI. Hence, both elevated ST-segment and Tp-e parameters were not only predictors of arrhythmia, but also prognostic value of MACE if they were mechanism-related, as in this study. So if Tp-e interval in ischemic-related leads mainly reflected TDR in acute ischemic state, it was increased following the change of ST segment in STEMI. This hypothesis explained and enriched the theory of the genesis of Tp-e interval and ST-segment elevation, just as the scheme in this discussion.

### 4.1 Study limitations

Some studies have reported poor inter- and intraobserver reproducibility of manual measurements of the ECG parameters.^[4,6]^ This shortfall applied to the measurement of the ST-segment and Tp-e as well in patients with STEMI. In addition, there has been some confusion as to how to measure these parameters with different configurations of the T-wave, especially for elevated ST-segment to assess T-wave markers. Recent experimental studies have clarified this, and we applied these methods in our measurements.^[4,22]^ However, we still excluded some cases difficult to identify the peak of the T-wave. Another limitation was that clinical results were deduced to cellular electrophysiologic mechanisms in this study. We should be cautious of the results extrapolated just by theory and inference for the conclusion of this study, but the inference for the meaning and utility of Tp-e interval was constructive and beneficial in pathophysiologic condition. A larger series and further investigation of in vitro and in vivo electrophysiological recording in STEMI could help confirm these results.

## 5 Conclusion

Elevated ST-segment and increased Tp-e had related electrophysiologic property to represent TDR in STEMI. Both STR and change of Tp-e parameters were not only predictors of arrhythmia, but also prognostic value of MACE in patients with STEMI after PCI.

## Author contributions

**Data curation:** Jialu Zhu.

**Formal analysis:** Lu Zhang, Chuanyu Gao.

**Funding acquisition:** Xianpei Wang, Chuanyu Gao.

**Investigation:** Xianpei Wang, Lu Zhang, Jialu Zhu, Xiaohang Yang.

**Methodology:** Xiaohang Yang.

**Project administration:** Xiaohang Yang.

**Resources:** Chuanyu Gao, Xiaohang Yang.

**Software:** Xiaohang Yang.

**Supervision:** Xianpei Wang, Chuanyu Gao.

**Writing – original draft:** Xianpei Wang, Chuanyu Gao.

## References

[1] Amaya N Nakano A Uzui H Relationship between microcirculatory dysfunction and resolution of ST-segment elevation in the early phase after primary angioplasty in patients with ST-segment elevation myocardial infarction. Int J Cardiol 2012;159:144–9. 21388694 10.1016/j.ijcard.2011.02.045

[2] Lonborg J Kelbaek H Holmvang L Comparison of outcome of patients with ST-segment elevation myocardial infarction and complete versus incomplete ST-resolution before primary percutaneous coronary intervention. Am J Cardiol 2016;117:1735–40. 27062938 10.1016/j.amjcard.2016.03.009

[3] Tjandrawidjaja MC Fu Y Westerhout CM Resolution of ST-segment depression: a new prognostic marker in ST-segment elevation myocardial infarction. Eur Heart J 2012;31:573–81. 10.1093/eurheartj/ehp494 19952006

[4] Castro Hevia J Antzelevitch C Tornes Barzaga F Tpeak-Tend and Tpeak-Tend dispersion as risk factors for ventricular tachycardia/ventricular fibrillation in patients with the Brugada syndrome. J Am Coll Cardiol 2006;47:1828–34. 16682308 10.1016/j.jacc.2005.12.049 PMC1474075

[5] Yan GX Joshi A Guo D Phase 2 reentry as a trigger to initiate ventricular fibrillation during early acute myocardial ischemia. Circulation 2004;110:1036–41. 15302777 10.1161/01.CIR.0000140258.09964.19

[6] Haarmark C Hansen PR Vedel-Larsen E The prognostic value of the Tpeak-Tend interval in patients undergoing primary percutaneous coronary intervention for ST-segment elevation myocardial infarction. J Electrocardiol 2009;42:555–60. PMID 19643432. 19643432 10.1016/j.jelectrocard.2009.06.009

[7] Tatlisu MA Ozcan KS Gungor B Can the T-peak to T-end interval be a predictor of mortality in patients with ST-elevation myocardial infarction? Coron Artery Dis 2014;25:399–404. 24618985 10.1097/MCA.0000000000000101

[8] Arteyeva NV Goshka SL Sedova KA What does the T(peak)-T(end) interval reflect? An experimental and model study. J Electrocardiol 2013;46:296e1–8. 23473669 10.1016/j.jelectrocard.2013.02.001

[9] Antzelevitch C Sicouri S Di Diego JM Does Tpeak-Tend provide an index of transmural dispersion of repolarization? Heart rhythm 2007;4:1114–6. 17675094 10.1016/j.hrthm.2007.05.028 PMC1994816

[10] Desmet WJ Mesotten LV Maes AF Relation between different methods for analysing ST segment deviation and infarct size as assessed by positron emission tomography. Heart 2004;90:887–92. 15253961 10.1136/hrt.2003.012955 PMC1768374

[11] Wang X Wu S Gao C Tpeak-Tend dispersion as a predictor for malignant arrhythmia events in patients with vasospastic angina. Int J Cardiol 2017;249C:61–5. 10.1016/j.ijcard.2017.07.093 29121758

[12] Tian C Song J He D Predictive value of mean platelet volume/platelet count for prognosis in acute myocardial infarction. Int Heart J 2018;59:286–92. 29563382 10.1536/ihj.17-212

[13] Chen J Zhang H Zhang W Correlated regression feature learning for automated right ventricle segmentation. IEEE J Transl Eng Health Med 2018;6:1800610. 30057864 10.1109/JTEHM.2018.2804947 PMC6061487

[14] Mugnai G Benfari G Fede A Tpeak-to-Tend/QT is an independent predictor of early ventricular arrhythmias and arrhythmic death in anterior ST elevation myocardial infarction patients. Eur Heart J Acute Cardiovasc Care 2016;5:473–80. 26228447 10.1177/2048872615598616

[15] Parchure N Batchvarov V Malik M Increased QT dispersion in patients with Prinzmetal's variant angina and cardiac arrest. Cardiovasc Res 2001;50:379–85. 11334842 10.1016/s0008-6363(00)00290-x

[16] Antzelevitch C Fish J Electrical heterogeneity within the ventricular wall. Basic Res Cardiol 2001;96:517–27. 11770069 10.1007/s003950170002

[17] Kanters JK Haarmark C Vedel-Larsen E T(peak)T(end) interval in long QT syndrome. J Electrocardiol 2008;41:603–8. 18822425 10.1016/j.jelectrocard.2008.07.024

[18] Zhao X Xie Z Chu Y Association between Tp-e/QT ratio and prognosis in patients undergoing primary percutaneous coronary intervention for ST-segment elevation myocardial infarction. Clin Cardiol 2012;35:559–64. 22740086 10.1002/clc.22022 PMC6652422

[19] Di Diego JM Antzelevitch C Acute myocardial ischemia: cellular mechanisms underlying ST segment elevation. J Electrocardiol 2014;47:486–90. 24742586 10.1016/j.jelectrocard.2014.02.005 PMC4116460

[20] Li RA Leppo M Miki T Molecular basis of electrocardiographic ST-segment elevation. Circ Res 2000;87:837–9. 11073877 10.1161/01.res.87.10.837

[21] Badri M Patel A Yan G Cellular and ionic basis of J-wave syndromes. Trends Cardiovasc Med 2015;25:12–21. 25446046 10.1016/j.tcm.2014.09.003

[22] Rivard L Roux A Nault I Predictors of ventricular arrhythmias and sudden death in a quebec cohort with brugada syndrome. Can J Cardiol 2016;32:1355.e1–7. 10.1016/j.cjca.2016.03.012 27378596

